# Ebola Virus Antibodies in Fruit Bats, Bangladesh

**DOI:** 10.3201/eid1902.120524

**Published:** 2013-02

**Authors:** Kevin J. Olival, Ariful Islam, Meng Yu, Simon J. Anthony, Jonathan H. Epstein, Shahneaz Ali Khan, Salah Uddin Khan, Gary Crameri, Lin-Fa Wang, W. Ian Lipkin, Stephen P. Luby, Peter Daszak

**Affiliations:** Author affiliations: EcoHealth Alliance, New York, New York, USA (K.J. Olival, A. Islam, S.J. Anthony, J.H. Epstein, P. Daszak);; Commonwealth Scientific and Industrial Research Organisation Australian Animal Health Laboratory, Geelong, Victoria, Australia (M. Yu, G. Crameri, L.-F. Wang);; Duke–National University of Singapore Graduate Medical School, Singapore (L.-F. Wang);; Chittagong Veterinary and Animal Sciences University, Chittagong, Bangladesh (S.A. Khan);; International Centre for Diarrhoeal Disease Research, Bangladesh, Dhaka, Bangladesh (S.U. Khan, S.P. Luby);; Columbia University, New York, New York, USA (W.I. Lipkin)

**Keywords:** bats, fruit bats, Chiroptera, Ebola virus, filovirus, viruses, Rousettus leschenaultii, natural reservoir, serology, zoonoses, Bangladesh

## Abstract

To determine geographic range for Ebola virus, we tested 276 bats in Bangladesh. Five (3.5%) bats were positive for antibodies against Ebola Zaire and Reston viruses; no virus was detected by PCR. These bats might be a reservoir for Ebola or Ebola-like viruses, and extend the range of filoviruses to mainland Asia.

Filoviruses are zoonotic pathogens that cause episodic, lethal, hemorrhagic outbreaks among humans and nonhuman primates and case-fatality rates up to 80% (1). The family *Filoviridae* contains 2 genera: *Marburgvirus*, which contains Marburg virus (MARV), and *Ebolavirus*, which contains 4 viruses: Zaire Ebola virus (ZEBOV), Sudan Ebola virus, Reston Ebola virus (REBOV), and Côte d’Ivoire Ebola virus, and 2 tentative species (Bundibugyo Ebola virus and Lloviu Ebola virus) (2,3). Pathogenicity varies among Ebola viruses, from ZEBOV, which is highly lethal in humans, to REBOV, which causes disease in pigs and macaques but asymptomatically infects humans.

Despite their role in human disease, natural reservoirs of filoviruses have remained elusive for decades. Reports suggest that bats (Order Chiroptera) are the primary natural hosts, including Old World insectivorous bats (genera *Rhinolophus* and *Miniopterus*) and frugivorous bats (family *Pteropodidae*). Fruit bats of the genus *Rousettus* have been implicated as a reservoir of filoviruses in Africa (4–7) and REBOV in the Philippines (8). Lloviu Ebola virus was detected in *Miniopterus schreibersii* insectivorous bats from Spain and appears to cause pathologic changes in this species but is not known to infect humans (2). These studies point to a wide, and still poorly described, geographic distribution for viruses of the family *Filoviridae* in chiropteran hosts. We screened bats of several species from Bangladesh for Ebola virus infection to determine whether the geographic range of this virus extends to southern Asia.

## The Study

We captured and sampled 276 bats (141 *Rousettus leschenaultii* bats, 75 *Cynopterus* spp. bats, 59 *Megaderma lyra* bats, and 1 *Macroglossus sobrinus* bat) during April 2010–March 2011 from the Faridpur, Rajbari, Lalmonirhat, and Comilla Districts in Bangladesh. All bats were identified to species in the field, except *Cynopterus* spp. bats, because of cryptic diversity in this group; we are awaiting genetic species confirmation. Bats were captured in mist nets near roosts or at feeding sites and were handled in accordance with the Tufts University (Medford, MA, USA) Institutional Animal Care and Use Committee protocol (no. G2011-106).

We collected 50–800 µL of blood from brachial or cephalic veins of each bat, and diluted it 1:4 with phosphate-buffered saline in the field before serum was separated, as described (9). We also collected throat, urine/urogenital, and fecal swab specimens, which were placed in 750 µL of NucliSENS lysis buffer (bioMérieux, Marcy l’Etoile, France). All samples were collected in cryovials, placed in liquid nitrogen in the field, and maintained at −80°C until testing. We recorded morphologic measurements, weight, sex, age, and body condition and collected a wing biopsy specimen before releasing animals at capture sites.

We screened serum samples for IgG against REBOV and ZEBOV by using ELISA and Western blotting at the Commonwealth Scientific and Industrial Research Organisation Australian Animal Health Laboratory Biocontainment Facility (Geelong, Victoria, Australia). To inactivate potentially infectious agents, serum samples were heated at 56°C for 20 min before shipment. All samples were screened by using a 1:1 mixture of purified recombinant nucleoproteins (0.2 mg/mL) of REBOV and ZEBOV (R + Z ELISA), which were expressed in an *Escherichia coli* vector that contained a histidine tag (10,11).

Potentially positive serum cutoff values were determined to be >0.454 for the R + Z ELISA by using maximum-likelihood estimation, gamma distribution, and 95% risk for error (7). Potentially positive serum samples were tested by ELISA against each nucleoprotein independently to confirm reactivity and by Western blotting against nucleoproteins of Reston and Zaire virus strains as described (10). Serum samples were tested at a dilution of 1:50. Endpoint titrations with an optical density >3× the background reading were determined for serum samples positive against REBOV and ZEBOV antigens individually.

Total nucleic acids were extracted from samples (urine/urogenital, fecal, and throat swab specimens) by using the easyMAG NucliSENS platform (bioMérieux) at Columbia University (New York, NY, USA). Samples were tested for filovirus RNA (RNA polymerase gene) by using a consensus PCR protocol validated to amplify 19 diverse filovirus strains. This PCR has a sensitivity of 50–500 RNA copies with synthetic transcripts and has been further validated with blood samples (12).

Fifteen (11%) of 141 *R. lescehnaulti*, 6 (8%) of 75 *Cynopterus* spp., and 4 (7%) of 56 *M. lyra* bats were potentially positive after initial screening. Five (3.5%) of 141 (95% CI 1.5%–8.0%) *R. leschenaultii* bats were confirmed as seropositive after testing by ELISAs and Western blotting (Table 1). Bats were sampled during the breeding season; 21 (62%) of 34 sampled female *R. leschenaultii* bats were pregnant and 8 (23%) of 34 carried pups. We sampled 3× as many males as females; all 5 confirmed virus-positive animals were healthy adult males (Table 2). All 698 throat, urine/urogenital, and fecal samples were virus negative by PCR (Table 2). All confirmed seropositive samples except 1 (April 2010–042) reacted more strongly to Zaire virus antigens than Reston virus antigens (Table 1). Similarly, 2 samples (April 2010–057 and SB0311–059) showed higher reactivity to ZEBOV by Western blotting, and other samples were equally reactive to REBOV.

**Table 1:** Ebola virus serologic assay results for bats, Bangladesh, 2010–2011*

Year, specimen no.	Age of bat	Sex of bat	Species or control	ELISA OD (endpoint titration)				Western blot	
				R + Z	R	Z		R	Z
2010									
Rab691/d0	ND	ND	Negative control	0.138	0.116	0.097		–	–
April 2010–001	A	F	Negative control (*Rousettus leschenaultii*)	0.215	0.117 (50)	0.058 (50)		–	–
April 2010–002	A	F	Negative control (*R. leschenaultii*)	0.092	0.096	0.059		–	–
Rab691/EboV-N	ND	ND	Positive control	**2.303**	**1.72**	**1.23**		**++**	**++**
Monkey/EboV	ND	ND	Positive control	**1.753**	**0.676**	**0.445**		NT	NT
April 2010–042	A	M	*R. leschenaultii*	**1.512**	**0.511 (400)**	0.07 (50)		+	+
April 2010–057	A	M	*R. leschenaultii*	**0.684**	0.072 (50)	**0.477 (800)**		+	++
66 additional negative	ND	ND	*R. leschenaultii*	<0.60	–	–		NT	NT
2011									
Rab691/d0	ND	ND	Negative control	0.165	0.116	0.145		–	–
SB0311–115	A	F	Negative control (*Megaderma lyra*)	0.515	0.074	0.083		–	–
SB0311–117	A	F	Negative control (*M. lyra*)	0.775	0.075	0.072		–	–
Rab691/ REboV-N	ND	ND	Positive control	**1.598**	**1.123**	**1.106**		**++**	**++**
SB0311–001	A	M	*R. leschenaultii*	**0.494**	0.213 (50)	**0.538 (100)**		**+**	**+**
SB0311–004	A	M	*R. leschenaultii*	**0.557**	0.152 (50)	**0.497 (100)**		+	+
SB0311–059	A	M	*R. leschenaultii*	**0.757**	0.079 (50)	**0.816 (400)**		–	++
SB0311–016	A	F	*R. leschenaultii*	**0.542**	0.182 (100)	**0.367 (400)**		NT	NT
67 additional negative	ND	ND	*R. leschenaultii*	<0.60	NT	NT		NT	NT
55 additional negative	ND	ND	*M. lyra*	<0.775	NT	NT		NT	NT
75 negative	ND	ND	*Cynopterus* sp.	<0.595	NT	NT		NT	NT
1 negative	A	M	*Macroglossus sobrinus*	<0.256	NT	NT		NT	NT

*Values in **boldface** are positive results. OD, optical density; R + Z, ELISA using a 1:1 mixture of recombinant nucleoproteins of Reston and Zaire Ebola viruses; R, Reston Ebola virus ELISA; Z, Zaire Ebola virus ELISA; ND, not determined;; A, adult; –, negative; ++, strongly positive; NT, not tested; +, positive.

**Table 2:** Bat specimen results for filovirus by PCR and Ebola virus by serologic analysis, Bangladesh, 2010–2011*

Bat species, sex, and sample type	No. positive/ no. tested
*Cynopterus* spp., n = 75, 43 M, 32 F	
Feces swab	0/74
Throat swab	0/75
Serum	0/75
Urine/urogenital swab	0/39
*Macroglossus sobrinus*, n = 1, 1 M	
Feces swab	0/1
Throat swab	0/1
Serum	0/1
Urine/urogenital swab	0/1
*Megaderma lyra*, n = 56, 23 M, 33 F	
Feces swab	0/56
Throat swab	0/56
Serum	0/56
Urine/urogenital swab	0/50
*Rousettus leschenaultii*, n = 141, 106 M, 34 F, 1 ND	
Feces swab	0/141
Throat swab	0/140
Serum	5/141
Urine/urogenital swab	0/58
Total	5/971

*ND, sex not determined.

## Conclusions

Our study provides evidence of Ebola virus infection in wildlife from mainland Asia and corroborates the observation that filoviruses are harbored across a much larger geographic range then assumed (2). Preferential reactivity to ZEBOV suggests exposure to an Ebola virus that is distinct from REBOV, the only filovirus currently found in Asia. We consider the likelihood of cross-reactivity with MARV as low because there is only a 35% aa identity between nucleoprotein genes of REBOV/ZEBOV and MARV. However, we cannot rule out co-infection with multiple filoviruses.

Seroprevalence found in this study is consistent with that found in another study (4). However, other studies of *Rousettus* spp. bats have reported higher values (e.g., 7%–20% and 8% of *R. aegyptiacus* bats seropositive for MARV and ZEBOV, respectively) (6,7), and 5 (31%) of 16 *R. amplexicaudatus* bats seropositive for REBOV (8). These differences might have been caused by poor specificity of the assay if this virus is novel, an artifact of low volume of blood collected, the potential that other species may have greater roles as reservoirs than *Rousettus* spp. in Bangladesh, or timing of sampling. *R. leschenaultii* bats have a large range (China to India) (13); and more detailed studies of virus ecology and diversity are warranted to better understand their role as a potential reservoir of zoonotic disease agents.

We demonstrated that serologic and virus surveys of bats can be informative for identifying potential virus hosts. Previous studies amplified ZEBOV nucleic acid from bat feces (14). We also screened bat feces to identify potential routes of virus excretion, which is useful when the route of exposure from bats to humans is known. A short interval for Ebola virus shedding by reservoir hosts and an inverse relationship between viremia and antivirus titer probably explain our negative PCR results for seropositive bats. Failure to detect filovirus nucleic acid might reflect our relatively small sample size, low virus prevalence, or use of a PCR that has low sensitivity for filoviruses circulating in Bangladesh.

In Bangladesh, human outbreaks of Nipah virus have been linked to drinking date palm sap contaminated with bat excreta, presumably from *Pteropus giganteus* bats (15). *R. leschenaultii* bats and other small fruit bat species visit date palm trees 10× more frequently than *Pteropus* spp. bats (15). This finding could indicate potential transmission of filoviruses or any other novel viruses that *R. leschenaultii* bats carry. It also highlights the need for more research to understand this ecologic system and for better implementation of low-cost barriers to reduce bat–human contact during periods of date palm harvesting (15).
